# Use of reagent test kits and fentanyl test strips among electronic music festival attendees in Colorado: prevalence, barriers, and behavior in response to drug checking

**DOI:** 10.1186/s12954-025-01181-4

**Published:** 2025-04-02

**Authors:** Cianna J. Piercey, Thomas E. Schlechter, Devin Henry, Mikayla Allen-Collins, Riley Ahern, Joseph Cameron, Bradley T. Conner, Jeffrey G. Snodgrass, Hollis C. Karoly

**Affiliations:** 1 Department of Psychology, Colorado State University, Fort Collins, CO 80521 USA; 2 Department of Psychiatry, University of Colorado School of Medicine, Denver, CO 80262 USA

**Keywords:** Polysubstance use, Drug checking, Fentanyl test strips, Reagent test kits, Music festivals, Electronic dance music, Harm reduction

## Abstract

**Background:**

Polysubstance use is common at electronic dance music (EDM) events and hazards associated with polysubstance use may be exacerbated when people who use drugs are unaware of the contents of their drug sample. Reagent test kits (RTK) and fentanyl test strips (FTS) are two efficacious drug checking tools that people who use drugs might use to protect themselves from risks associated with contamination, adulteration, and misrepresentation of unregulated substances. In the current study, we aimed to (1) characterize the use of RTK and FTS among attendees of a 4-day music festival in Colorado and (2) qualitatively capture perceived barriers to using RTK and FTS within festival settings.

**Methods:**

We surveyed 227 music festival attendees on their use of drug checking tools (i.e., RTK and FTS) and behavior in response to drug checking. We also collected qualitative data on perceived barriers of using RTK and FTS within a festival setting using survey-based open-ended text response questions.

**Results:**

The percentage of participants having ever used RTK and FTS was 75.3% and 66.5% respectively. When asked how often participants ensure their drugs are tested prior to consumption, participants responding “always” or “most of the time” was 54.4% for use of RTK and 59.4% for use of FTS. Additionally, 60.8% of participants reported that they had never consumed a drug that reagent tested differently than expected and 87.9% of participants reported that they had never consumed a drug that tested positive for fentanyl. Perceived barriers to using RTK and FTS within a festival setting encompassed the following themes: (1) accessing testing materials (2) environmental or ecological barriers (3) legal concerns (4) social dynamics (5) lack of education/training and (6) limits of individual drug checking tools.

**Conclusions:**

RTK and FTS appear to empower festival attendees in the U.S. to make informed decisions related to their substance use. However, there is a critical need to reduce barriers associated with drug checking for this at-risk population.

## Introduction

Electronic dance music (EDM) represents an 11.8-billion-dollar industry globally, with club and festival sales estimated to account for over half of revenues [1]. The United States currently has the second largest concentration of Spotify EDM listeners in the world behind Germany [1] and routinely hosts major EDM festivals, such as the Electronic Daisy Carnival Las Vegas, which attracted over half a million attendees in 2024 [2]. Simultaneous polysubstance use is prevalent at EDM events [3, 4] and involves the use of multiple substances such that their effects overlap [5]. Prior research indicates that simultaneous polysubstance use is associated with increased incidence of adverse drug-related outcomes due to potential interactions between substances [6–8].

### Risks of polysubstance use within an unregulated drug market

Exacerbating risks associated with polysubstance use, individuals attending EDM events face a significant concern regarding the consumption of adulterated or contaminated drugs [9, 10]. Drug adulteration involves the intentional addition of ingredients to a drug sample that may be pharmacologically inert (e.g., sugar) or active (e.g., levamisole) [11, 12], while drug contamination refers to the unintentional mixing of substances during production and distribution processes. Due to the unregulated nature of purchasing party drugs from a criminalized market, individuals also run the risk that any drug purchased is an entirely different substance than what they intended to consume [13]. Additionally, festival contexts may be particularly susceptible to circulation of poor-quality drugs (i.e., contaminated, adulterated, and/or falsely advertised) given that sales often occur within an isolated purchase at events, without rapport established between drug seller and consumer [14].

The conditions described above amplify risks associated with simultaneous polysubstance use through multiple mechanisms. First, people who use drugs may unknowingly engage in simultaneous polysubstance use if their drug sample is adulterated or contaminated with other pharmacologically active substances. Additionally, uncertainty surrounding the content of one’s drug sample(s) may preclude people who use drugs from engagement with polysubstance use harm reduction recommendations. For example, one simultaneous polysubstance use protective strategy is to start with less of each substance than one would typically consume if only using one substance [4]. However, uncertainty about drug sample content (and thus uncertainty surrounding dosing parameters) may preclude engagement with such recommendations. Finally, people who use drugs may inadvertently consume a dangerous or even fatal combination of drugs when drug sample content is unknown.

### Harm reduction strategies

To mitigate harms associated with polysubstance use within the context of an unregulated and potentially toxic drug supply, several community and individual-level harm reduction strategies have been identified. For example, community or event-level harm reduction practices described in the EDM event literature include strategies such as the provision of designated “chill out” zones and peer support services (e.g., Zendo Project, Dance Safe, Kosmicare), as well as the distribution of evidenced-based educational materials and harm reduction supplies [15–17]. Individual-level practices include, but are not limited to, purchasing drugs from known sources, taking breaks while dancing, ensuring adequate hydration, exercising caution when combining drugs, carrying naloxone, and avoiding using drugs alone [4, 18–20]. Drug checking, or the process of analyzing the contents of an unknown drug sample, is a harm reduction practice that can be implemented at both the community (e.g., provision of drug checking services) and individual level. However, formal drug checking services remain rare in the U.S. and are seldom provided at EDM events, likely due to variations in the legality of drug checking equipment across states [21]. Thus, Individuals attending U.S.-based events must often rely on informal or self-checking practices in order to test their drugs. Reagent test kits (RTK) and fentanyl test strips (FTS) are two efficacious drug testing tools that may be used for these purposes.

### Drug checking

RTK involves colorimetric analysis of a drug sample to confirm the presence of an individual’s desired drug compound [22]. Procedures for the use of RTK involve placing a small amount of the drug to be tested on a clean, non-porous surface (e.g., ceramic plate) and carefully adding a drop of a reagent onto the sample. Individuals are then directed to watch for color changes and compare the observed chemical reaction to the expected color change for the substance being tested. For example, when testing a suspected 3–4 methylenedioxy-methamphetamine (MDMA) sample with Marquis, the expected color reaction is purple/black. However, if a different color reaction were to be observed, one might conclude that the sample contains a different compound (e.g., yellow/red is the expected reaction with Marquis when a sample contains amphetamine). To gain further clarity, the process may then be repeated with other types of reagents if they are available. For example, if the expected reaction is obtained with the Marquis reagent, one might then use a Mandelin or Simon’s reagent to cross-check against the reaction observed with Marquis. Thus, reagents are often sold in kits that include multiple tests, though tests may also be purchased individually.

FTS are single-use immunoassay tests that provide a binary result indicating the presence or absence of fentanyl (and some fentanyl analogues) in a drug sample [23]. While FTS instructions may vary depending on the substance being tested and the planned route of administration, procedures for the use of FTS generally involve diluting one’s drug sample into water (either a portion or the full amount), dipping the test strip into the solution, and then waiting for one (positive for fentanyl) or two lines (negative for fentanyl) to appear on the strip. After interpreting the test result, one may then need to reconstitute their drug sample if they tested their full batch of drugs (e.g., when testing drugs that will later be insufflated or smoked). Notably, testing one’s full drug sample is often considered the most ideal way to use FTS due to a phenomenon colloquially known as the “chocolate chip cookie” effect. Specifically, fentanyl may not be evenly distributed within one’s drug sample and may not be detected if only a portion of the sample is tested.

### Barriers and limitations of RTK and FTS

RTK and FTS are both generally considered rapid, cost efficient, and interpretable without the high-level scientific knowledge that may be implicated in other drug checking technologies [24, 25]. However, these tools are imperfect and confer a lower level of discrimination than more sophisticated tests, highlighting the importance of educating festival attendees on the limitations of these tools.

For instance, RTK cannot be used to determine the potency or purity of a drug sample, and reagent testing renders the portion of the sample being tested nonviable for later consumption [25]. Drug samples that contain a mixture of substances may also be more challenging to accurately identify, and color reactions may vary depending on drug concentration or salt form [24]. Further, RTK may not exist for certain drugs, particularly novel psychoactive substances [25], which are commonly used in the dance scene [26]. FTS were originally developed to detect fentanyl metabolites in urine but have since been adapted for off-label drug checking purposes [25]. One limitation of FTS is that they may be unable to detect all fentanyl and fentanyl analogues present in a drug sample. Additionally, FTS are only able to produce a binary positive or negative result for fentanyl and cannot detect other potential adulterants or contaminants that may be present, or determine drug sample potency. Some brands of FTS may also produce false positives if certain stimulants or cutting agents are present in the sample [24].

Prior work has also identified barriers and challenges to engagement with drug checking tools within real-world settings, such as difficulty accessing testing supplies and interpreting test results [27, 28]. Additionally, logistical concerns have been posed by people who use drugs, including lack of access to a private testing location, the time it takes to test, unwillingness to sacrifice a portion of one’s drug sample, and concerns regarding the legality of testing supplies [27–29]. However, data on barriers to the use of drug checking tools within U.S. dance settings is limited and may differ from those encountered in other drug use contexts [14].

### Use of RTK and FTS in the U.S. dance music scene

Research on the use of RTK and FTS among EDM event attendees in the U.S. is currently limited. Prior work examining the use of RTK in this population focused exclusively on testing of MDMA or ecstasy [10, 30]. To our knowledge, no studies published at the time of this writing have examined U.S. EDM event attendees’ personal use of RTK to test drugs other than ecstasy (e.g., cocaine, ketamine) and there are currently no published studies examining the use of FTS in this population. Further, little is known about barriers to the personal/informal use of RTK and FTS within EDM event settings, such as festivals.

The current study leveraged a mixed methods field study design to conduct in person surveys of drug checking behaviors among attendees of a 4-day electronic music festival in the U.S. Specifically, we sought to characterize use of RTK and FTS in this population (e.g., prevalence of use, specific drugs tested, behavior in response to drugs testing differently than expected) and qualitatively capture perceived barriers to using RTK and FTS within festival settings.

## Methods

### Participants

Participants included 227 attendees of Sonic Bloom Music Festival in Rye, Colorado (Table 1). Participants were eligible for the study if they were between the ages of 18–65 and were not visibly intoxicated at the time of recruitment. The sample was predominantly comprised of men (49.5%) and women (41.0%), with a mean age of 28.2 years old (SD = 5.6). Most participants identified their race as White (78%) and their ethnicity as White or European American (63.4%), generally reflecting the racial and ethnic makeup of the state of Colorado. Notably, over 40% of the sample identified as a sexual minority. The study received approval from the Institutional Review Board at Colorado State University.

**Table 1:** Respondent characteristics of Sonic Bloom music festival attendees, June 2023, Rye, Colorado

Characteristics	*N*	%
	*M (SD)*	*Min/Max*
Age	28.2 (5.6)	*18/55*
Gender		
Agender	3	1.4
Gender fluid	9	4.1
Gender queer	3	1.4
Gender questioning	1	0.5
Man	110	49.5
Woman	91	41.0
Non-binary	4	1.8
Prefer not to answer	1	0.5
Transgender		
Yes	5	2.2
No	215	96.4
Prefer not to answer	3	1.3
Ethnicity		
Arab, Middle Eastern, or North African	15	6.6
Asian or Asian American	19	8.4
Black or African American	10	4.4
Hispanic or Latino	25	11.0
Native American or Alaska Native	8	3.5
Native Hawaiian or Other Pacific Islander	3	1.3
White or European American	144	63.4
Not listed	6	2.6
Race		
Asian	11	4.8
Black	8	3.5
Indigenous, Aboriginal, or First Nations	3	1.3
Latino or Hispanic	27	11.9
Middle Eastern	7	3.1
White	177	78.0
Not listed	2	0.9
Sexual Orientation		
Straight or heterosexual	133	58.6
Lesbian	3	1.3
Gay	5	2.2
Bisexual	52	22.9
Pansexual	22	9.7
Sexually fluid	10	4.4
Queer	10	4.4
Demisexual	8	3.5
Asexual	2	0.9
Questioning	4	1.8
I use a different term	1	0.4
Prefer not to answer	6	2.6
Education		
Less than high school	2	0.9
High school diploma or GED	32	14.7
Some college	49	22.5
Associates degree or technical certification	17	7.8
Bachelor’s degree	94	43.1
Master’s degree	19	8.7
Doctoral degree	5	2.3
Household Income		
$0-$9,999/yr	12	5.5
$10,000-$19,999/yr	14	6.4
$20,000-$29,999/yr	23	10.5
$30,000-$39,999/yr	30	13.6
$40,000–49,999/yr	29	13.2
$50,000-$59,999/yr	29	13.2
Over $60,000/yr	83	37.7

Note. M, mean. SD, standard deviation

### Procedure

Recruitment was conducted informally by study staff using convenience sampling methods (i.e., no systematic approach to participant selection was employed). Recruitment occurred on days two through four of the festival, with the first day allocated for travel to the festival and staff camp set-up. Participants were approached while they were tailgating at the event campground, roughly between the hours of 11am to 6pm. During each day of recruitment, the study team divided to cover different sections of the campground, with the aim of ensuring broad representation across the site. When approached by study staff, attendees were invited to participate in an anonymous self-administered survey on substance use and harm reduction, which took approximately 15 min to complete. All questions were delivered in a Qualtrics survey format, and no interviews were conducted as part of this study. Participants could complete the survey on their own devices using a QR code or use a study iPad if their device was uncharged or did not have service. As compensation, participants received a commemorative art print created by the first author (CJP) of this manuscript. Additionally, all individuals approached by study staff were offered free harm reduction supplies (e.g., naloxone, fentanyl test strips, safer snorting supplies), regardless of their decision to participate.

### Measures

#### Demographics

Participants were asked to provide their age, gender, sexual orientation, ethnicity, race, education level, and household income (Table 1).

#### Substance use

Participants reported their lifetime, past year, and event-specific use of 22 substances. Event-specific substance use included substances used on previous days of the festival and planned use for the remaining days. For example, those recruited on the third day of the festival reported their use on days one and two, and their planned use for days three and four. Detailed substance use data is presented in Table 2.

**Table 2:** Polysubstance use patterns among Sonic Bloom music festival attendees, June 2023, Rye, Colorado

Drugs endorsed	Lifetime		Past year		Day 1		Day 2		Day 3		Day 4	
	*N*	%	*N*	%	*N*	%	*N*	%	*N*	%	*N*	%
No use	0	0	0	0	45	19.8	37	16.3	35	15.4	29	12.8
Tobacco/nicotine	189	83.3	171	75.3	115	50.7	127	55.9	126	55.5	116	51.1
Alcohol	219	96.5	206	90.7	138	60.8	155	68.3	145	63.9	122	53.7
Cannabis	221	97.4	206	90.7	138	60.8	155	68.3	151	66.5	142	62.6
MDMA	212	93.4	175	77.1	34	15.0	77	33.9	106	46.7	38	16.7
LSD	212	93.4	161	70.9	27	11.9	46	20.3	91	40.1	24	10.6
Cocaine	192	84.6	144	63.4	55	24.2	62	27.3	61	26.9	48	21.1
Psilocybin	212	93.4	172	75.8	56	24.7	87	38.3	72	31.7	54	23.8
DMT	158	69.6	87	38.3	11	4.8	21	9.3	32	14.1	20	8.8
Peyote/mescaline	29	12.8	11	4.8	3	1.3	2	0.9	2	0.9	2	0.9
2C series	43	18.9	18	7.9	3	1.3	2	0.9	5	2.2	2	0.9
Ketamine	173	76.2	138	60.8	59	26.0	83	36.6	88	38.8	66	29.1
Nitrous oxide	156	68.7	107	47.1	50	22	55	24.2	55	24.2	41	18.1
Poppers	54	23.8	30	13.2	2	0.9	2	0.9	2	0.9	2	0.9
GHB	22	9.7	4	1.8	2	0.9	2	0.9	3	1.3	2	0.9
Kratom	83	36.4	36	15.9	5	2.2	6	2.6	6	2.6	6	2.6
Kava	65	28.6	28	12.3	3	1.3	3	1.3	4	1.8	3	1.3
Heroin	20	8.8	6	2.6	2	0.9	2	0.9	2	0.9	2	0.9
Fentanyl	15	6.6	8	3.5	2	0.9	2	0.9	2	0.9	2	0.9
Methamphetamine	40	17.6	12	5.3	3	1.3	3	1.3	3	1.3	3	1.3
Prescription pain killers	75	33.0	14	6.2	2	0.9	3	1.3	2	0.9	2	0.9
Prescription stimulants	117	51.5	62	27.3	12	5.3	15	6.6	14	6.2	12	5.3
Prescription anti-anxiety	97	42.7	31	13.7	6	2.6	11	4.8	10	4.4	9	4.0

#### Reagent testing

Participants were asked if they have ever tested their drugs with RTK, selecting from the following response options: (1) “Yes, I have personally tested my drugs with reagents” (2) “Yes, a harm reduction organization has tested my drugs with reagents” (3) “Yes, a friend or family member has tested my drugs with reagents” (4) “Yes, a dealer has tested my drugs with reagents” (5) “Yes, an entity not listed here has tested my drugs with reagents” and (6) “No, I have never tested my drugs with reagents”. Participants selecting any response other than “No, I have never tested my drugs with reagents” were asked to rate how frequently they ensure their drugs are reagent tested before use on a 5-point Likert scale (1 = Never, 5 = Always). Participants also indicated which drugs they have reagent tested and whether any test results differed from expectations. If a discrepancy was reported, participants were asked if they had consumed a drug despite unexpected test results. Finally, through a survey-based open-ended text-response question, participants were asked to describe any barriers they have experienced using RTK within a festival setting.

#### Fentanyl test strips

The items and branching logic for FTS mirrored those for reagent testing. Additionally, participants were asked to select from a list which of their drugs had tested positive for fentanyl using FTS.

### Analysis plan

To characterize engagement with RTK and FTS and behavior in response to drug checking, prevalence rates are reported as a percentage for each categorical variable (computed in IBM SPSS Statistics for Windows version 29). Open-ended questions on drug checking barriers (for both RTK and FTS) were thematically analyzed in Microsoft Excel and MAXQDA 24 by the first, second, and last author (CJP, TES, and HCK) following guidelines established by Braun and Clarke [31]. First, the coders thoroughly read through each participant response to familiarize themselves with the data. They then engaged in open-coding to generate an initial set of agreed upon codes, with a focus on identifying repetitions and similarities and differences across the data [32]. CJP and TES subsequently independently coded each participant response for both open-ended questions. They then engaged in discussion to resolve codes until 100% agreement was reached between coders, and they searched for and reviewed themes. Finally, they defined themes and selected exemplar responses (detailed below).

## Results

### Substance use

All participants reported lifetime and past year substance use. The most commonly used substances on any given day of the festival were alcohol (53.7–68.3%), cannabis (60.8–68.3%) and tobacco/nicotine products (50.7–55.9%). The next most commonly used substances were ketamine (26%), psilocybin (24.7%), and cocaine (24.2%) on day 1, psilocybin (38.3%), ketamine (36.6%), and MDMA (33.9%) on day 2, MDMA (46.7%), Lysergic acid diethylamide (LSD) (40.1%), and ketamine (38.8%) on day 3, and ketamine (29.1%), psilocybin (23.8%), and cocaine (21.1%) on day 4. Polysubstance Use Patterns are reported in Table 2.

### Reagent test kits

Regarding RTK use, 54.2% of participants reported personally testing their drugs, while 24.7% had never tested their drugs with reagents. Other responses included testing by friends or family (25.6%), dealers (22.9%), and harm reduction organizations (7%). Additionally, 0.9% shared that an entity not listed had tested their drugs with reagents. When asked about frequency of ensuring drugs are reagent tested before use, 27.2% of participants responded “always”, 27.2% responded “most of the time”, 10.7% responded “about half of the time”, 29.6% responded “sometimes”, and 5.3% of participants responded “never”. The most tested substances were MDMA (61.7%), cocaine (48.9%), ketamine (37%), and LSD (18.1%). Additionally, a minority of participants reported testing psilocybin (5.3%), DMT (4%), 2C series chemicals (4%), methamphetamine (3.5%), prescription anti-anxiety (2.6%), prescription stimulants (1.8%), and prescription pain killers (1.8%) with RTK. When participants were asked if their drug samples had ever tested differently than expected, 44.4% answered “yes” and 55.6% answered “no”. Additionally, when asked if they had ever consumed a drug that tested differently than expected (*N* = 74), 39.2% responded “yes” and 60.8% responded “no”. Patterns of RTK use are provided in Table 3.

**Table 3:** Reagent test kit use among Sonic Bloom music festival attendees, June 2023, Rye, Colorado

Survey Item	*N*	%
Have you ever tested your drugs with reagents? Select all that apply.		
Yes, I have personally tested my drugs with reagents	123	54.2
Yes, a harm reduction organization has tested my drugs with reagents	16	7.0
Yes, a friend or family member has tested my drugs with reagents	58	25.6
Yes, a drug dealer has tested my drugs with reagents	52	22.9
Yes, an entity not listed here has tested my drugs with reagents	2	0.9
No, I have never tested my drugs with reagents	56	24.7
Which drugs have you tested before with reagents?		
MDMA/Ecstasy/Molly	140	61.7
LSD	41	18.1
Cocaine	111	48.9
Psilocybin	12	5.3
DMT	9	4.0
Peyote/mescaline	2	0.9
2C series	9	4.0
Ketamine	84	37
GHB	1	0.4
Heroin	2	0.9
Fentanyl	7	3.1
Methamphetamine	8	3.5
Prescription pain killers	4	1.8
Prescription stimulants	4	1.8
Prescription anti-anxiety	6	2.6
How often do you ensure your drugs are reagent tested prior to consumption?		
Never	9	5.3
Sometimes	50	29.6
About half the time	18	10.7
Most of the time	46	27.2
Always	46	27.2
When using reagents, have you ever found that your drug sample tested differently than you were expecting?		
No	95	55.6
Yes	76	44.4
After using reagents, have you ever consumed a drug that did not test as expected?		
No	45	60.8
Yes	29	39.2

### Fentanyl test strips

Approximately half of the sample (50.7%) reported personally testing their drugs with FTS, whereas 13.7% reported testing by a friend or family member, 9.3% reported testing by a dealer, 6.6% by a harm reduction organization, 0.4% by another entity, and 33.5% of the sample reported never having used FTS. Regarding the frequency of testing drugs for fentanyl before consumption, 30.7% of participants responded “always”, 28.7% responded “most of the time”, 6% responded “about half of the time”, 30% responded “sometimes”, and 4.7% responded “never”. The most tested substances were again MDMA (48.5%), cocaine (42.7%), and ketamine (28.6%). Less commonly tested substances included DMT (4.4%), prescription anti-anxiety medications (4%), LSD (3.5%), psilocybin (2.6%), prescription painkillers (2.2%), prescription stimulants (1.8%), 2C series chemicals (1.3%), peyote/mescaline (0.4%), heroin (0.9%), methamphetamine (0.9%), and cannabis (0.4%). When asked if their drug sample had ever tested positive for fentanyl, 22% responded “yes” and 78% “no”. Participants indicated that the following drugs had tested positive for fentanyl: MDMA (7.5%), cocaine (5.7%), ketamine (3.1%), prescription pain killers (1.8%), prescription stimulants (1.3%), methamphetamine (0.9%), heroin (0.9%), prescription anti-anxiety (0.9%), LSD (0.4%), peyote/mescaline (0.4%), 2C series chemicals (0.4%) and cannabis (0.4%). Additionally, 12.1% of participants reported an instance in which they consumed a drug that tested positive for fentanyl, whereas 87.9% reported never consuming a substance that tested positive. Patterns of FTS use are reported in Table 4.

**Table 4:** Fentanyl test strip use among Sonic Bloom music festival attendees, June 2023, Rye, Colorado

Survey Item	*N*	%
Have you ever tested your drugs with fentanyl test strips? Select all that apply.		
Yes, I have personally tested my drugs with fentanyl test strips	115	50.7
Yes, a harm reduction organization has tested my drugs with fentanyl test strips	15	6.6
Yes, a friend or family member has tested my drugs with fentanyl test strips	31	13.7
Yes, a drug dealer has tested my drugs with fentanyl test strips	21	9.3
Yes, an entity not listed here has tested my drugs with fentanyl test strips	1	0.4
No, I have never tested my drugs with fentanyl test strips	76	33.5
Which drugs have you tested before with fentanyl test strips?		
MDMA/Ecstasy/Molly	110	48.5
LSD	8	3.5
Cocaine	97	42.7
Psilocybin	6	2.6
DMT	10	4.4
Peyote/mescaline	1	0.4
2C series	3	1.3
Ketamine	65	28.6
GHB	1	0.4
Heroin	2	0.9
Methamphetamine	2	0.9
Prescription pain killers	5	2.2
Prescription stimulants	4	1.8
Prescription anti-anxiety	9	4.0
Cannabis	1	0.4
How often do you ensure your drugs are fentanyl tested prior to consumption?		
Never	7	4.7
Sometimes	45	30.0
About half the time	9	6.0
Most of the time	43	28.7
Always	46	30.7
Have your drugs ever tested positive for the presence of fentanyl?		
No	117	78.0
Yes	33	22.0
Which of the following drugs have you had test positive for fentanyl?		
MDMA/Ecstasy/Molly	17	7.5
LSD	1	0.4
Cocaine	13	5.7
Psilocybin	0	0
DMT	0	0
Peyote/mescaline	1	0.4
2 C series	1	0.4
Ketamine	7	3.1
GHB	0	0
Heroin	2	0.9
Methamphetamine	2	0.9
Prescription pain killers	4	1.8
Prescription stimulants	3	1.3
Prescription anti-anxiety	2	0.9
Cannabis	1	0.4
Have you ever consumed a drug after it tested positive for fentanyl?		
No	29	87.9
Yes	4	12.1

### Drug checking barriers

**Fig. 1:**
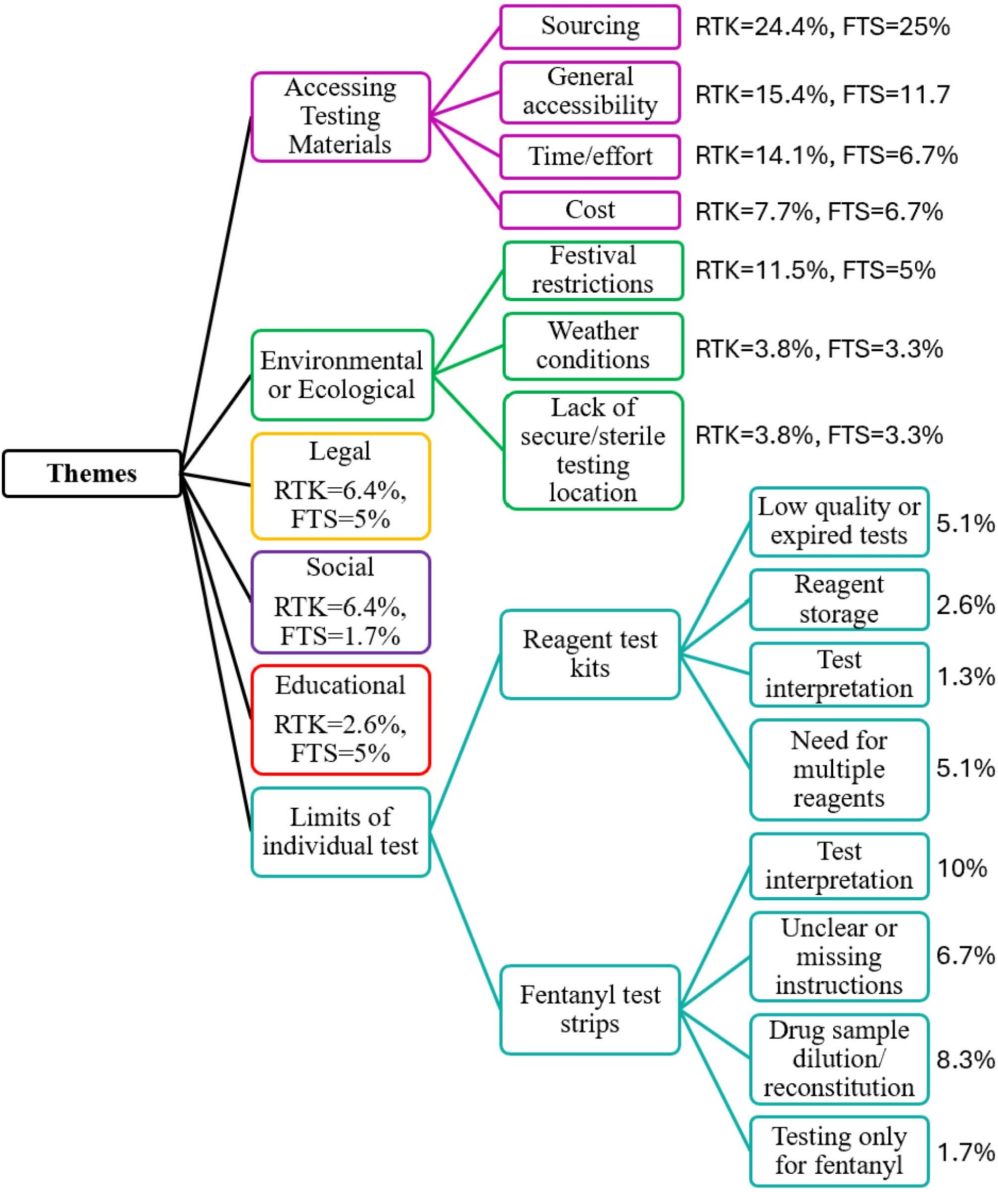
Themes of drug checking barriers reported by Sonic Bloom music festival attendees, June 2023, Rye, Colorado

Perceived barriers to drug checking in a festival setting (Fig. 1) encompassed six themes: (1) accessing testing materials (2) environmental and ecological barriers (3) legal concerns (4) social dynamics (5) lack of education/training and (6) limits of the drug checking tool. Participants who reported on barriers to using RTK was N = 78 and participants who reported on barriers to using FTS was N = 60.

#### Theme 1: accessing testing materials

Accessibility barriers included financial cost (RTK = 7.7%, FTS = 6.7%), challenges with sourcing of drug checking tools (RTK = 24.4%, FTS = 25%), the time and effort it takes to perform drug checking procedures (RTK = 14.1%, FTS = 6.7%), and general accessibility (i.e., participants named accessibility as a barrier, but did not elaborate in their response; RTK = 15.4%, FTS = 11.7%).

*“Monetary cost. Lack of access (for buying tests personally).”* Man, 23 years old

*“No access to free test strips in my entire county”* Woman, 28 years old.

*“Availability at festivals or not having enough time to order before the festival.”* Woman, 27 years old.

*“Cost*,* the process.”* Woman, 39 years old.

*“Time*,* takes a while.”* Woman, 23 years old.

*“More places to find strips rather than one booth or stand”* Man, 26 years old.

#### Theme 2: environmental and ecological barriers

The festival environment posed some barriers to using test kits even when participants had the materials available. Some participants reported that the weather conditions at festivals (e.g. wind) and the nature of conducting testing outside was an environmental barrier to drug checking (RTK = 3.8%, FTS = 3.3%). Festival restrictions also posed a barrier (RTK = 11.5%, FTS = 5.0%), with participants noting the lack of drug checking services and prohibition of drug checking supplies at many events. Additionally, participants shared that the lack of a secure and sterile testing location at festivals made drug checking more difficult in these spaces (RTK = 3.8%, FTS = 3.3%).

*“Weather makes testing difficult for powders.”* Agender person, 28 years old.

*“Logistics of doing it in an area exposed to the weather”* Woman, 34 years old

*“Some festivals do not allow testing kit sales or the entrance of them- this should be allowed to curate a better experience”* Man, 25 years old.

*“I have to rely on myself to bring the kits so if I don’t have them the fact that many festivals don’t allow the sales of kits is a struggle.”* Woman, 25 years old.

*“Clean test environment”* Man, 27 years old.

*“Not having a secure place to test and land-owners who don’t support drug test groups”* Man, 23 years old.

#### Theme 3: legal concerns

In addition to restrictions imposed by festivals, some participants reported concerns about the legality of drug checking tools and fear surrounding potential legal repercussions related to possession of testing materials (RTK = 6.4%, FTS = 5.0%).

*“I think they are technically illegal to have and are considered paraphernalia.”* Man, 25 years old.

*“feeling like I’ll get in trouble for using one of the tests”* Woman, 27 years old.

*“In some states it’s a felony to carry test kits”* Man, 31 years old.

#### Theme 4: social dynamics

Social dynamics (RTK = 6.4%, FTS = 1.7%) were identified as another barrier to drug checking, with some participants reporting that intergroup dynamics and “peer pressure” could make testing difficult. Some participants also reported that drug sellers could be hesitant about testing and another shared that testing results were not adequately communicated to them.

*“Social dynamics. I’m always adamant that we not take or use anything from strangers without testing it first but my partner is more comfortable with having a good feeling from the person and that’s it. We’ve had fights about it and sometimes it’s easier to just go along with it. I don’t use until it’s tested in those cases*,* but it makes me uncomfortable that my partner will.”* Woman, 34 years old.

*“Some dealers can be hesitant about letting their stuff be tested.”* Man, 31 years old.

*“Peer pressure and non-presence of testers”* Man, 30 years old.

*“Lack of communication if something has tested badly”* Woman, 30 years old.

#### Theme 5: lack of education and training

Lack of education or awareness about drug checking and lack of training in how to perform drug checking procedures was also reported by some participants (RTK = 2.6%, FTS = 5.0%).

*“Don’t know how to use*,* friend normally does it”* Woman, 23 years old.

*“Not enough exposure to resources*” Man, 24 years old.

*“I didn’t know how to do it correctly”* Woman, 25 years old.

#### Theme 6: limitations of the drug checking tool

Challenges related to the design of drug checking tools, test quality, and procedures involved in drug checking were also noted, with barriers varying between RTK and FTS.

##### RTK Specific Barriers

RTK limitations included needing to properly store reagents to keep them cool (2.6%), concerns about test accuracy and interpretation (1.3%), and encountering low quality or expired tests (5.1%). Additionally, participants reported that the need for multiple reagents across multiple substances created another barrier to engagement with RTK (5.1%).

*“Not enough different testers to test if the substance tests clean on merc*,* marquis*,* mandolin”* Man, 32 years old.

*“No full panel tests. Sometimes difficult to have multiple kits for separate substances at once.”* Gender not shared, 32 years old.

*“Keeping reagents cool is another barrier”* Agender person, 28 years old.

*“Access to tests for some drugs*,* like ketamine”* Man, 26 years old.

*“Some cheap tests are inaccurate”* Gender queer person, 35 years old.

##### FTS Specific Barriers

Barriers related to the use of FTS included difficulty and uncertainty surrounding the interpretation of test results (10.0%) and the fact that FTS only test for fentanyl despite the effort involved in testing (1.7%). Participants also reported challenges understanding instructions included with FTS or shared that FTS were sometimes distributed without instructions (6.7%). Additionally, participants reported that diluting and reconstituting their drug sample in a festival setting created another substantial barrier to use of FTS (8.3%), particularly for drugs where insufflation is a primary route of administration (e.g., ketamine, cocaine).

*“The faded negative stripe can sketch me out sometimes. There might be a very faded negative stripe but technically that counts as negative. Also the differences in substance dissolved concentrations are not always clear in the instructions. For instance the testing concentration for meth/mdma is different than cocaine”* Man, 32 years old.

*“Can’t boil cocaine down easily to test at campsite”* Woman, 27 years old

*“I want to test our whole amount but we can’t dissolve it to test it and dehydrate it in a festival setting”* Agender person, 28 years old.

## Discussion

Risks of polysubstance use may be exacerbated when individuals are unaware of the contents of their drug sample, particularly given the ongoing polydrug overdose crisis in the U.S. and the presence of an unregulated and potentially toxic drug supply [33, 34]. This study aimed to explore the use of RTK and FTS among attendees of a 4-day music festival in Colorado, including attendees’ perceived barriers to engagement with drug checking.

Most participants reported at least one prior experience of checking their drugs with RTK or FTS. However, one quarter of those who used RTK and one fifth of those who used FTS had never personally tested their drugs. Among those who had never personally tested their drugs, participants primarily relied on friends to test, potentially due to reported accessibility barriers such as financial cost, difficulty sourcing supplies, and lack of education/training. For example, it may be more feasible for one member of a social circle to acquire drug checking materials and test for everyone in the group, particularly if friends are sharing from the same batch of drugs. However, aside from accessing drug testing through a harm reduction organization, self-testing could be the most optimal way to ensure that testing procedures are adequately followed. Thus, there may be a need for interventions that encourage U.S. festival attendees to test their own drugs when possible, particularly considering that many U.S.-based festivals do not permit harm reduction organizations to conduct on-site drug checking. Indeed, less than one tenth of the sample reported an instance of their drugs being checked by a harm reduction organization, likely due to such restrictions [8, 14].

Participants most commonly tested MDMA, cocaine, and ketamine, potentially given awareness that powders and tablets have a higher risk of adulteration or contamination compared to plant or fungi materials like cannabis or mushrooms [11]. Despite this, some participants still reported testing plant material, with one individual reporting a suspicion that their cannabis tested positive for fentanyl. While there is a case report of a patient in treatment for opioid use disorder testing positive for fentanyl via urinalysis (despite purportedly only consuming cannabis) [35], there is little evidence to support the intentional adulteration of cannabis with fentanyl at this time. Although accidental contamination could occur, participants in this study were not asked to describe specific testing methods followed or how they interpreted their results. Thus, while it is challenging to confirm the accuracy of this report, it appears unlikely, particularly given that the study was conducted in Colorado, where cannabis is legal and regulated.

Among those who reported a time when their drugs tested differently than expected with RTK, three out of five participants stated they had never consumed a drug that yielded unexpected test results, whereas two out of five reported that they had. However, since participants were not asked to specify how often this occurred, it remains unclear whether such instances were isolated or regularly occurring. It’s also important to acknowledge that consuming a substance that tests differently than anticipated does not necessarily reflect a lack of behavior modification, though this wasn’t inquired about in the current study. Some participants may have adjusted their dosage or employed other harm reduction strategies [3, 18, 20, 36, 37] after discovering the substance was different from what they expected. As for participants who reported a time when their drugs tested positive for fentanyl with FTS, most participants reported they did not consume the substance, while roughly one out of 10 participants stated that they did. This finding highlights the crucial role of FTS in empowering festival attendees to make informed choices regarding their substance use, with most participants choosing not to consume a substance testing positive for fentanyl.

Barriers to drug checking in U.S. festival settings included accessing testing materials (i.e., sourcing, cost, and time/effort involved in testing), environmental or ecological challenges (i.e., weather conditions, festival restrictions, lack of secure/sterile testing location), legal concerns, social dynamics, lack of education and training, and individual limitations of RTK and FTS. Foremost, these findings underscore a clear need for increased distribution of free and low-cost drug checking supplies at festivals, particularly at events in which drug checking services are not permitted on-site, as is the case at most festivals in the U.S. Festivals may also reduce barriers by allowing attendees to bring their own drug checking supplies into the festival and by providing attendees with clean and weather-protected drug checking zones. Ultimately, U.S. festivals allowing harm reduction services at events would reduce the burden of self-checking, ensuring that festival attendees have access to qualified drug checkers on site. However, it is notable that people who use drugs may in some cases prefer to self-check their drugs [38], particularly given fears surrounding legality identified in the present study and prior work [39].

To address individual limitations of RTK and FTS, there is a need for ongoing concerted efforts aimed at improvement of drug checking technology, ensuring that tools are user-friendly and have clear instructions. While some studies have found high levels of FTS acceptability among people who use drugs, with participants reporting ease following instructions and interpreting test results [23, 40, 41], some participants of the current study identified unclear or missing instructions and difficulty interpreting test results as a barrier to the use of FTS. One potential explanation for this finding is the prevalence of participants in the current study using FTS to test MDMA. Instructions for testing MDMA often vary across harm reduction organizations in terms of recommended testing method and drug sample dilution protocols, potentially contributing to confusion. For example, some harm reduction organizations recommend diluting MDMA in a teaspoon of water, whereas others recommend diluting MDMA in up to half a cup of water. Additionally, individuals testing MDMA may experience greater difficulty in their interpretation of test results given potentially growing awareness that these drugs can produce false positives with FTS [42].

### Actionable recommendations to mitigate drug checking barriers

To overcome barriers related to the use of RTK and FTS within U.S. festival settings, we have devised several actionable recommendations based on study findings. Specifically, the recommendations listed below were developed within the context of settings where legal or other concerns prevent festivals from providing direct drug checking services to attendees:

- Station multiple harm reduction booths throughout festival grounds, including within festival campgrounds.
  - Deploy volunteers to circulate festival grounds and inform attendees of booth locations, as well as distribute testing materials when permitted to do so.
- If possible, offer free or low-cost testing supplies, such as through a sliding scale model.
  - Ensure any testing materials distributed include clear and specific instructions for performing testing procedures and interpreting test results.
  - Supply additional resources such as clean containers, surfaces, and measuring tools for accurate drug testing.
  - Provide attendees with weather-protected, designated drug-checking zones.
- When provision of supplies is not possible, allow attendees to bring their own drug checking supplies into the festival and connect attendees with off-site drug checking resources prior to the event.
- Offer on-site training in the use of RTK and FTS, ideally at multiple timepoints throughout the festival. Festivals may also consider distributing educational materials to attendees prior to the event (e.g., through social media or event websites).
- Ensure affordable or free access to ice to support proper reagent storage.
- Provide intervention materials to counteract stigma and peer influence surrounding drug checking.
- Offer psychoeducation on drug checking legality specific to the festival location to ensure attendees are informed of relevant laws.

### Limitations and future directions

This study has several limitations, including its cross-sectional design, reliance on participant self-report, and the relatively homogenous demographic of participants. Findings may also have limited generalizability beyond festival settings. Given that participants were recruited from a single EDM festival in Colorado, findings may not represent the experiences of individuals attending festivals across other geographical locations or music genres. This study also used convenience sampling methods and thus may have been susceptible to selection bias. Additionally, we failed to track the number of attendees who were invited to participate and the number of attendees who did not meet inclusion criteria (i.e., aged 18–65 and not visibly intoxicated at the time of participation), therefore we were unable to calculate the participant response rate or exclusion rate. Further, participants were not asked to report on variables such as frequency of substance use, dosing patterns, route of administration, or age of first use.

Future research should aim to replicate these findings with a more diverse sample and explore polysubstance use patterns and use of drug checking tools in other high-risk recreational contexts. Future research would also benefit from employing probability sampling methods (e.g., systematic random sampling, stratified random sampling) to increase sample representativeness [43] and from keeping detailed records of the recruitment process that would allow for the calculation of participant response rates and exclusion rates. Additionally, application of longitudinal methods (e.g., daily diary) would provide a more nuanced understanding of drug checking behaviors and how they evolve over time. This could include examining protective strategies for polysubstance use that go beyond simply avoiding the consumption of a misrepresented or contaminated drug, such as dose modification or carrying naloxone. Future research should also explore decision making processes following drug checking in further depth and encourage participants to provide detailed accounts of the drug checking procedures they followed. Further, future studies may benefit from inquiring about festival attendees’ awareness of and engagement with other drug checking technologies (e.g., Gas Chromatography Mass Spectroscopy, High-Performance Liquid Chromatography, Fourier-Transform Infrared Spectroscopy).

## Conclusions

RTK and FTS appear to empower festival attendees in the U.S. to make informed decisions related to their substance use, highlighting the importance of testing drugs prior to consumption. However, there is a critical need to reduce barriers associated with drug checking at festivals, which include access to testing materials, environmental and ecological barriers, legal concerns, social dynamics, lack of education and training, and limitations of RTK and FTS technologies.

## Data Availability

Data and materials are available from the corresponding author upon reasonable request.

## Abbreviations

EDM: Electronic dance music
RTK: Reagent test kits
FTS: Fentanyl test strips
MDMA: 3–4 methylenedioxy-methamphetamine
LSD: Lysergic acid diethylamide
